# Pharmacokinetic interaction of curcumin and glibenclamide in diabetic rats

**DOI:** 10.14202/vetworld.2015.508-511

**Published:** 2015-04-19

**Authors:** P. R. Sakunthala Devi, A. Gopala Reddy, G. S. Rao, C. S. V. Satish Kumar, G. Boobalan

**Affiliations:** 1 Department of Veterinary Pharmacology and Toxicology, College of Veterinary Science, Sri Venkateswara Veterinary University, Tirupati - 517 502, Andhra Pradesh, India; 2 Department of Veterinary Pharmacology & Toxicology, NTR College of Veterinary Science, Sri Venkateswara Veterinary University, Tirupati - 517 502, Andhra Pradesh, India

**Keywords:** curcumin, glibenclamide, pharmacokinetics, CYP3A4

## Abstract

**Aim:**

The aim was to assess the pharmacokinetic (PK) interaction of curcumin and glibenclamide (GL) in diabetic rats.

**Materials and Methods:**

Sprague-Dawley rats induced with diabetes were divided into 2 groups of six rats in each. Group I: GL (6 mg/kg *po* once daily) treatment in diabetic rats and group 2: Curcumin (50 mg/Kg *po* once daily) + GL (dose as above) in diabetic rats. Blood samples were collected at pre-determined time intervals for kinetic analysis after the first and last oral dosing of GL for single and multiple dose studies, respectively. Plasma samples were assayed for GL concentration by high-performance liquid chromatography and PK parameters were analyzed.

**Results:**

The half-life (t_1/2_) and mean residence time (MRT) of GL were significantly increased in curcumin pre-treated rats as compared to GL alone in single and multiple dose studies. Similarly, the V_dss_ was significantly increased in curcumin pre-treated rats in single dose study as compared to GL alone treated group, but no significant difference was observed in multiple dose kinetics.

**Conclusion:**

The study revealed higher values (t_1/2,_ MRT and V_dss_) of GL in curcumin pre-treated group due to the inhibitory effect of curcumin on intestinal CYP3A4.

## Introduction

Diabetes mellitus is a metabolic disorder featured by hyperglycemia and alterations in carbohydrate, fat and protein metabolism associated with absolute or relative deficiency of insulin secretion and/or insulin action. The incidence of the disorder is significantly increasing worldwide [1].

Glibenclamide (GL) is a second-generation sulphonylurea, which has been widely used in the management of Type-2 diabetes [2]. GL kinetics showed a rapid and complete absorption (90-100 %) from the gastrointestinal tract [3]. GL is mainly metabolized by isozyme CYP3A4 [4]. The metabolites *viz*., 4-trans hydroxyl and 3-cis hydroxyl derivatives contribute no significant hypoglycemic action.

Curcumin is a low molecular weight polyphenol derived from turmeric [5]. It has a wide spectrum of biological functions such as antidiabetic, anti-inflammatory, immunomodulatory, and neuroprotective functions [6]. Herbal medicines that modulate intestinal and hepatic CYPs can alter the bioavailability and clearance of co-administered drugs [7]. Curcumin showed a competitive type of inhibition towards CYP1A2, CYP3A4, whereas a non-competitive inhibition was observed with respect to CYP2D6 and CYP2C9 [8]. Hence, there was a possibility of curcumin for the metabolic inhibition of GL, which is metabolized by CYP3A4 microsomal liver enzymes [9-11].

The aim of this study was to highlight the clinical interactions between herbal remedy (curcumin) and prescribed drug (GL).

## Materials and Methods

GL was administered as the suspension in freshly prepared 2% w/v gum acacia. Pure standard Curcumin was administered in olive oil as a vehicle.

### Ethical approval

Male albino rats of Sprague Dawley strain weighing around 200-250 g were procured from National Institute of Nutrition (NIN), Hyderabad. The experimental protocol was approved by the Institutional Animal Ethics Committee.

### Experimental design

The study was carried out on 12 diabetic rats that were randomly divided into two groups comprising 6 rats in each group for single and multiple dose kinetics of GL on day 1 following curcumin pre-treatment and at the end of continuous daily administration of curcumin and GL for 2 months.

Group 1: GL (6 mg/kg *po* once daily) treatment in diabetic rats [12]

Group 2: GL (as above) + curcumin (50 mg/Kg *po*) once daily in diabetic rats [13]

Rats were induced diabetes by intraperitoneal injection of streptozotocin @ 40 mg/kg body weight. The rats were provided with glucose water for 24 h to prevent hypoglycemia. After 72 h, blood samples were collected for glucose estimation. Rats with blood glucose value of >250 mg/dl (72 h after streptozotocin administration) were included in the study (n=6). Treatment protocols were initiated from day 2 post-confirmation of diabetes (day 5 post-streptozotocin administration) and were continued for 8 weeks.

### Blood collection for pharmacokinetic (PK) studies

Blood (approximately 500 µl) was collected from retro-orbital plexus at 0, 5, 10, 20, 40, and 90 min, and 2 h, 4 h, 8 h, 12 h, and 24 h after oral administration of GL in required dose into heparinized containers and plasma was separated by centrifugation at 3000 RPM for 15 min and stored at −20°C till analysis.

### High-performance liquid chromatography (HPLC) assay of GL in rat plasma

GL was extracted from plasma samples by liquid-liquid extraction technique. Methanol was added to plasma at a ratio of 1:1 and thoroughly mixed by vortexing for 30 s. The mixture was then centrifuged at 5000 rpm for 10 min. The clear supernatant thus obtained was transferred into microcentrifuge tubes following filtration through 0.2 µ HN nylon membrane filter. A 20 μl of filtrate was injected manually into HPLC with dual wavelength UV detector (SPD-20A) and LC solution^®^ software for data analysis. A reverse phase C_18_ Column (250 ×4.5 mm, particle size 5±0.3µm, pore diameter 100±10 Å, Phenomenax^®^, (USA) served as stationary phase. Mobile phase consisted of 0.1% orthophosphoric acid: methanol: acetonitrile mixed at a ratio of (20:50:30) volumes. The flow rate of mobile phase was maintained @ 1 ml per minute. The detection wavelength was set at 240 nm (UV). The plasma samples were analyzed for 8 min at room temperature. There were no interfering peaks in the chromatogram at the retention time (R_t_ = 6.00±0.82) of GL.

### PK analysis

The plasma concentration-time profile of GL of each animal was used to determine its PK. The PK data of GL was subjected to compartmental analysis. Following oral administration, the plasma levels of GL were described by non-compartmental analysis of data. Different PK parameters were analyzed using the software PK solver 2.0 [14].

### Statistical analysis

Single and multiple dose PK data were statistically analyzed by applying non-parametric test while other data were subjected to statistical analysis by applying one-way ANOVA using Statistical Package for Social Sciences (SPSS) version 15.0. Differences between means were tested using Duncan’s multiple comparison test and significance level was set at 0.05.

## Results

**Figure-1:**
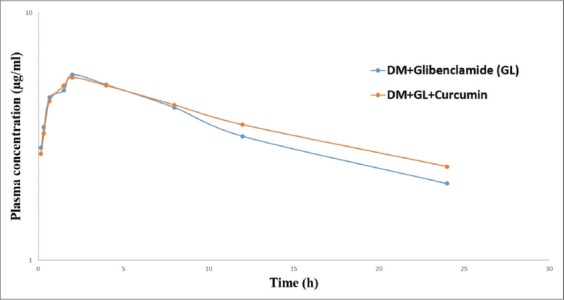
Plasma concentration of glibenclamide (µg/ml) following pre-treatment with curcumin in diabetic rats during single dose pharmacokinetic studies.

Single dose administration of GL in Group 1 resulted in detectable concentration of the drug (2.85±0.02 mg/ml) at 10 min and the peak plasma level of 5.62±0.05 mg/ml was achieved at 2 h (Table-1 and Figure-1). Elimination rate constant, elimination half-life, AUC0-t, mean residence time (MRT), Vdss, and CLβ were 0.05/h, 10.03±0.05 h, 83.49±0.84 mg/ml h, 14.89±0.23 h, 1.54±0.01 L/kg and 0.07±0.00 L/kg/h, respectively (Table-2). Curcumin pretreated rats (Group 2) showed elimination rate constant, elimination half-life, AUC0-t, MRT, Vd(area), and CLβ were 0.04/h, 10.42±0.10 h, 87.86±1.94 mg/mlh, 18.80±0.83 h, 1.84±0.05 L/kg, and 0.07±0.00 L/kg/h, respectively (Table-2)

**Table-1:** Mean plasma concentration of GL (µg/ml) following pre-treatment with curcumin in diabetic rats during single dose PK studies.

Time (h)	DM+GL	DM+GL+Curcumin
0.16	2.85±0.02	2.69±0.04
0.33	3.45±0.03	3.25±0.05
0.66	4.54±0.06	4.39±0.11
1.5	4.85±0.07	5.07±0.08
2.0	5.62±0.05	5.48±0.07
4.0	5.12±0.04	5.08±0.10
8.0	4.14±0.04	4.24±0.07
12.0	3.17±0.03	3.53±0.11
24.0	2.04±0.04	2.39±0.10

Values are Mean±SE (n=6), SE=Standard error, GL=Glibenclamide, PK=Pharmacokinetic

**Table-2:** Pharmacokinetic parameters of GL following pre-treatment with curcumin in diabetic rats during single dose PK studies.

Parameter		DM+GL		DM+GL+Curcumin
Cmax (µg/ml)		5.63±0.05		5.48±0.07
Tmax (h)		2		2
Elimination rate constant (h^−1^)		0.05±0.00		0.04±0.00
T_1/2β_ (h)		10.03±0.05		10.42±0.10*
AUC _(0-t)_ (µg-h/ml)		83.49±0.84		87.86±1.94
MRT (h)		14.89±0.23		18.80±0.83*
V_d (area)_ (L/kg)		1.54±0.01		1.84±0.05*
CL_B_ (L/kg/h^−1^)		0.07±0.00		0.07±0.00

SE=Standard error, GL=Glibenclamide, PK=Pharmacokinetic,

* Significant variation at p≤0.05 Duncan’s multiple comparison test, values are mean±SE (n=6)

**Figure-2:**
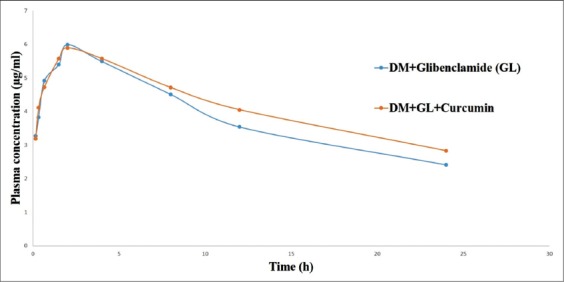
Plasma concentration of glibenclamide (µg/ml) following pre-treatment with curcumin in diabetic rats during multiple-dose pharmacokinetic study.

Multiple oral administration of GL (6 mg/kg) in rats resulted in detectable concentration of the drug (3.27±0.02 mg/ml) at 10 min and the peak plasma level of 5.99±0.05 mg/ml was achieved at 2 h (Table-3 and Figure-2). Elimination rate constant, elimination half-life, AUC_0-t_, MRT, V_dss,_ and CL_β_ were 0.04/h, 10.23±0.02 h, 92.41±0.84 mg/ml h, 16.60±0.24 h, 1.55±0.02 L/kg, and 0.07±0.00 L/kg/h respectively (Table-4). Curcumin pre-treated rats, detectable concentration of the drug was 3.19±0.03 mg/ml at 10 min and the peak plasma level of 5.89±0.12 mg/ml was achieved at 2 h (Table-3 and Figure-2). Elimination rate constant, elimination half-life, AUC_0-t_, MRT, V_dss,_ and CL_β_ were 0.034/h, 10.63±0.10 h, 99.82±2.02 mg/ml h, 21.89±1.03 h, 1.82±0.01 L/kg, and 0.06±0.00 L/kg/h, respectively (Table-4).

**Table-3:** Mean plasma concentration of GL (µg/ml) following pre-treatment with curcumin in diabetic rats during multiple dose PK studies.

Time (h)	DM+GL	DM+GL+Curcumin
0.16	3.27±0.02	3.19±0.03
0.33	3.82±0.03	4.11±0.07
0.66	4.91±0.06	4.72±0.23
1.5	5.4±0.07	5.57±0.11
2.0	5.99±0.05	5.89±0.12
4.0	5.49±0.04	5.57±0.14
8.0	4.51±0.04	4.71±0.10
12.0	3.5-4±0.03	4.05±0.11
24.0	2.41±0.04	2.83±0.11

Values are mean±SE (n=6), SE=Standard error, GL=Glibenclamide, PK=Pharmacokinetic

**Table-4:** Pharmacokinetic parameters of GL following pre-treatment with curcumin in diabetic rats during multiple dose PK studies.

Parameter	DM+GL	DM+GL+Curcumin
Cmax (µg/ml)	5.99±0.05	5.89±0.12
Tmax (h)	2	2
Elimination rate constant (h^−1^)	0.042±0.00	0.034±0.00
T_1/2β_ (h)	10.23±0.02	10.63±0.10*
AUC _(0-t)_ (µg-h/ml)	92.41±0.84	99.82±2.02
MRT (h)	16.60±0.24	21.89±1.03*
V_d (area)_ (L/kg)	1.55±0.02	1.82±0.01
CL_B_ (L/kg/h)	0.07±0.00	0.06±0.00

* Significant variation at P≤0.05 Duncan’s multiple comparison test, Values are Mean±SE (n=6), SE=Standard error, GL=Glibenclamide, PK=Pharmacokinetic

## Discussion

In this study, we investigated the PK interactions of curcumin with GL, which is metabolized by CYP3A4 microsomal liver enzymes. GL is metabolized by CYP3A4 and is a substrate for intestinal P-glycoprotein [15]. Curcumin could also give rise to drug interactions as it has been reported to inhibit both the function and expression of P-gp [16]. Several *in vitro* studies reported the inhibition of CYP 450s, especially CYP3A4, CYP1A2, and CYP2C9 by curcumin [8].

In single-dose study, there was a significant increase in the t_1/2β_ (18.80±0.83 h) and MRT (1.84±0.05 h) values in curcumin pre-treated rats when compared to GL alone treated group. Similarly, V_dss_ (1.82±0.01 L/kg) was significantly increased in curcumin pre-treated rats as compared to GL alone treated group. Similarly in multiple dose study, there was a significant increase in the t_1/2_ and MRT in curcumin pre-treated group, compared to GL used alone. GL is extensively metabolized by CYP3A4 and is also a substrate for P-gp. Organic anion transport proteins are reported to be responsible for transport of large number of endogenous and xenobiotic compounds across cell membranes [17].

The most useful term in PKs is the apparent volume of distribution at steady state or V_dss_. It gives an idea of the relative degree of drug binding in the blood and extravascular space. Increased V_dss_ may be due to increased penetration of drug which resulted in prolonged t_1/2_ and MRT [18].

The increase in t_1/2,_ MRT, and V_dss_ may be due to the inhibitory effect of curcumin on intestinal CYP34A in intestine and liver cells, thus decreasing the metabolism of GL.

## Conclusion

The present study concluded that curcumin pre-treatment to diabetic rats in single and multiple dose study increased the t_1/2,_ MRT, and V_dss_ of GL suggesting a synergistic PK profile.

## Authors’ Contributions

PRSD supervised the overall research work. PRSD, CSVSK, and GB performed the study. AGR and GSR participated in draft and revision of the manuscript. All authors read and approved the final manuscript.
